# Identification of a New Genetic Clade of Cowpea Mild Mottle Virus and Characterization of Its Interaction With Soybean Mosaic Virus in Co-infected Soybean

**DOI:** 10.3389/fmicb.2021.650773

**Published:** 2021-04-08

**Authors:** Zhongyan Wei, Chenyang Mao, Chong Jiang, Hehong Zhang, Jianping Chen, Zongtao Sun

**Affiliations:** State Key Laboratory for Managing Biotic and Chemical Threats to the Quality and Safety of Agro-products, Institute of Plant Virology, Ningbo University, Ningbo, China

**Keywords:** high-throughput sequencing, phylogenetic analysis, genetic variation, recombination, co-infection

## Abstract

Cowpea mild mottle virus (CPMMV; genus *Carlavirus*) can be a destructive pathogen of soybean but there is little information about its distribution on soybean in China. Here, we collected soybean plants with virus-like symptoms from 11 fields widely scattered within China, and used high-throughput sequencing to determine their virome. Most samples (8/11) were co-infected by the well-studied potyvirus soybean mosaic virus (SMV) and CPMMV, and the remaining three samples were singly infected with CPMMV. The near-complete genome sequences of the 11 CPMMV isolates were determined and phylogenetic analysis showed that they constituted a new genetic clade. One recombination event was detected among the CPMMV sequences, and the isolate CPMMV_JL_CC was identified as recombinant. In mechanical inoculation assays, co-infection by CPMMV and SMV resulted in an enhancement of disease symptoms, but decreased the expression level of the genomic RNAs and CP of CPMMV, without significantly affecting SMV accumulation. The interaction between these viruses needs further investigation.

## Introduction

Soybean [*Glycine max* (L.) Merr.], one of the most important oil and cash crops worldwide, provides protein-rich food and feed for humans and animals (Wilson, 2008). Many different pests and pathogens attack soybean crops causing significant losses in yield and quality (Widyasari et al., 2020) and some of the most serious pathogens are viruses (Wrather and Koenning, 2006). Soybean virus diseases typically cause 10–30% yield losses but losses of 50–100% have been reported from severe outbreaks (Hartman et al., 2011; Song et al., 2016). Soybean mosaic virus (SMV) is the most important virus infecting soybean worldwide but a wide variety of other viruses have been reported, including cucumber mosaic virus, cowpea mild mottle virus (CPMMV) and alfalfa mosaic virus (Hill and Whitham, 2014).

SMV, a member of the genus *Potyvirus* in the family *Potyviridae*, has a single-strand RNA genome that encodes a single large open reading frame (ORF) (Hajimorad et al., 2018; Gao et al., 2019). The translated polyprotein yields a series of multifunctional proteins through proteolysis, which are commonly named P1, HC-Pro, P3, PIPO (a product of slippage in the P3 coding sequence), 6K1, CI, 6K2, VPg, NIa-Pro, NIb, and CP (Hajimorad et al., 2018; Gao et al., 2019). SMV is naturally transmitted by aphids in a nonpersistent manner but can also be transmitted through infected seeds (Widyasari et al., 2020). Plants infected with SMV usually have mottling on their leaves, stem necrosis and are stunted. Strains of SMV have been recognized based on their genomic similarity and the response of different soybean cultivars. In this way, isolates were classified into seven strains (G1–G7) in the United States (Cho and Goodman, 1979), and more recently into 22 strains (SC1–SC22) in China (Li et al., 2010; Wang et al., 2013).

CPMMV is another economically important virus that causes serious damage to soybean production particularly in Brazil (Zanardo et al., 2014a,b). It was first reported from cowpea in Ghana and subsequently found in other leguminous crops across the world (Brunt and Kenten, 1973; Zanardo and Carvalho, 2017). CPMMV is a positive-strand RNA virus that belongs to the genus *Carlavirus* in the family *Betaflexiviridae*. The genome of CPMMV (7.8–8.9 kb) encodes six ORFs (ORF 1-6) that are translated into the corresponding proteins: Replicase including the RNA-dependent RNA polymerase (RdRp; ORF1), triple gene block proteins (TGBs; ORFs2-4), coat protein (CP; ORF5) and a nucleic-acid-binding protein (NABP; ORF6) (Zanardo and Carvalho, 2017). CPMMV is transmitted by the whitefly *Bemisia tabaci* in a nonpersistent manner, and the predominant symptoms in soybean are mosaic, stem necrosis and dwarfing (Muniyappa, 1983; Zanardo and Carvalho, 2017). The genomes of CPMMV isolates infecting soybean have been characterized from Brazil, Ghana and India (Brunt and Kenten, 1973; Yadav et al., 2014; Zanardo et al., 2014a,b). Recently, we found CPMMV infecting soybean in Anhui province in China (Wei et al., 2020) but the distribution, importance and biological characteristics of CPMMV in China are largely unknown.

In this study, we collected soybean plants with virus-like symptoms from different regions in China and used high-throughput sequencing (HTS) to investigate the presence of viruses. All the samples contained CPMMV, often in co-infections with SMV, and these CPMMV isolates formed a single clade, distinct from previously reported isolates from elsewhere in the world. Plants co-infected with CPMMV and SMV had enhanced disease symptoms, but had reduced expression levels of the genomic RNA and CP of CPMMV compared to those infected by CPMMV alone.

## Materials and Methods

### Virus Isolates

Leaves of soybean plants with typical virus-like symptoms were collected from 11 fields among six soybean-growing provinces of China (Jilin, Shandong, Henan, Anhui, Jiangsu and Hubei) during September 2019. Pools of leaves from three to five symptomatic plants in each field were wrapped in plastic bags and placed in dry ice.

### RNA Sequencing

Total RNA was extracted from each sample by TRIzol Reagent (Invitrogen, United States). Approximately 10 μg of total RNA was used for transcriptome sequencing. The RNA sequencing was performed by Zhejiang Tiangen (Company, Hangzhou, China). The final cDNA library was constructed using the TruSeq Stranded mRNA Library Prep Kit (Illumina, United States) and sequenced on an Illumina HiSeq 4000 (LC Sciences, United States).

### Analysis of Sequencing Reads

The reads were generated through the Illumina paired-end RNA-seq approach, the average insert size for the paired-end library was 300 ± 50 bp. Prior to assembly, the low-quality reads were removed. Clean data were assembled using Trinity software (version 2.8.5). The assembled contigs were first compared with Barcode of Life Data (BOLD) Systems^1^, and searched against NCBI virus RefSeqs^2^ using a BlastX algorithm with a cutoff E-value of 10^–5^.

### RT-PCR Detection

About 1.5 μg of total RNA used in the RNA sequencing assay was reversely transcribed to cDNA using a reverse transcription kit (Tiangen Company, Beijing, China). The cDNA (0.5 μL) was amplified by PCR using KOD-FX (Toyobo, Osaka, Japan), with the following conditions: 94°C for 3 min, 34 cycles of 98°C for 10 s, 58°C for 30 s, and 72°C for 2 min. Primers used for amplifying sequences of specific viruses (CPMMV and SMV) are listed in Supplementary Table 2. PCR products were separated on a 1.2% agarose gel.

### Phylogenetic Analysis

The nearly complete genome sequences of isolates obtained in this study and reported isolates retrieved from NCBI^3^ were aligned by MUSCLE method in MEGAX. Phylogenetic trees were constructed using Maximum-Likelihood (ML) methods using the best-fitting model: GTR + G + I (General Time Reversible + Gama Distributed With Invariant Sites). To check the reliability of the constructed trees, the bootstrap test with 1,000 bootstrap replications was used. For the CPMMV phylogenetic tree construct, Indian citrus ringspot virus (*Mandarivirus*, *Alphaflexiviridae*) was used as the outgroup. Turnip mosaic virus (*Potyvirus, Potyviridae*) was used as the outgroup for the SMV phylogenetic tree construct.

### Recombination Analysis

Putative recombination events amongst CPMMV isolates and SMV isolates were identified using the recombination detection program RDP5 (Martin et al., 2020), and evaluated using different methods: RDP, GENECONV, BOOTSCAN, MaxiChi, Chimera, SiScan, and 3Seq. Alignments of nucleotide sequences produced in MEGAX were run in RDP5 (P-value cut-off of 0.05). Only recombination events that were detected by three or more methods were considered.

### Mechanical Transmission Assay

For inoculation of soybean plants, sap from each of the 11 symptomatic plant pools was used to inoculate a susceptible soybean variety (Jiunong 9). Each inoculum was prepared from 1 g of symptomatic field plant leaves homogenized with 10 mL of 0.01 M phosphate-buffered saline, pH 7.0. After mixing with carborundum, inoculation was performed manually before the trifoliate leaves emerged and the inoculum finally rinsed with tap water. Plants inoculated with phosphate-buffered saline were used as controls. Inoculated plants were grown at 25–28°C (16 h light/8 h dark) in an incubator for 7–10 days, and classified into four phenotype classes based on symptoms of 12 plants per inoculation: symptomless, mosaic, crinkling, stem necrosis. Three independent experiments were conducted to provide data for statistical analysis, Values are means ± Standard Deviation (*SD*).

### RNA Extraction and RT-qPCR

Total RNA was extracted from young trifoliate leaves using TRIzol reagent (Invitrogen, United States). About 1.5 μg of total RNA was reversely transcribed to cDNA using a reverse transcription kit (Tiangen Company, Beijing, China). Real-time PCR was conducted using ChamQ^TM^ SYBR qPCR Master Mix (Low ROX Premixed) by an ABI7900HT Sequence Detection System (Applied Biosystems, Carlsbad, CA, United States). The RT-qPCR conditions were as follows: 95°C for 4 min; 40 cycles of 95°C for 10 s and 60°C for 30 s. The relative expression levels of genes were determined using the 2^–ΔΔC (t)^ method (Livak and Schmittgen, 2001). The soybean Actin11 gene was used as an internal control. Three biological and two technical replicates were conducted to determine gene expression. The RT-qPCR primer sequences used in this study are listed in Supplementary Table 2.

### Western Blot

Approximately 200 mg soybean leaves were homogenized in 0.3 mL sodium dodecylsulfate (SDS) lysis buffer (100 mm Tris-HCl, pH 6.8, 10% SDS and 2.0% β -mercaptoethanol). The crude extracts were centrifuged at 12,000 × g for 10 min at room temperature, and the resulting supernatant (8 μL per sample plus 2 μL 5 × SDS loading buffer) was electrophoresed in 10–12% SDS-PAGE gels. Western blot analysis was done as previously reported (Zhang et al., 2019). Proteins were transferred to polyvinylidene difluoride (PVDF, Millipore, United States) membranes using the Trans-Blot Turbo transfer system (Bio-Rad, United States). Polyclonal rabbit anti-CPMMV-CP and anti-SMV-CP (at 1:3,000 dilution, synthesized by Huabio, China) were used to detect the respective viruses.

## Results

### Occurrence of SMV and CPMMV in China

**FIGURE 1:**
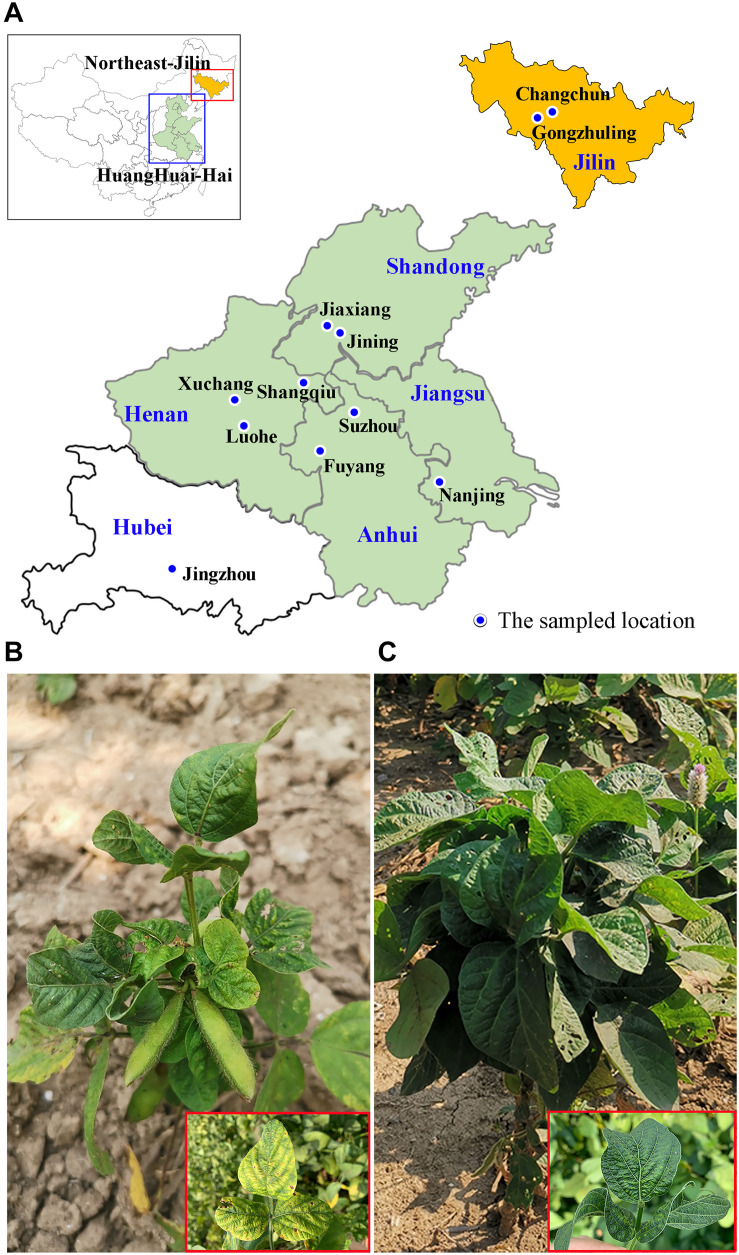
The geographical locations of the symptomatic soybean samples collected in China and the symptoms on leaves of the sampled plants. **(A)** Map of China (top left corner) showing the study area (red and blue rectangular box). The 11 sampled fields are marked on the enlarged abridged map. **(B,C)** Leaves of soybean plants displaying typical yellow mosaic **(B)** and crinkling **(C)** virus-like symptoms.

Soybean samples with mosaic and crinkling symptoms on their leaves were collected from 11 locations in six provinces (Jilin, Shandong, Henan, Anhui, Jiangsu and Hubei) in the main soybean producing region of China (Figures 1A–C and Table 1). After extraction of total RNA, high-throughput sequencing (HTS) yielded more than 25 M paired-end reads from each sample (ranging from 25.38 to 54.50 M). BLASTX analysis of the assembled contigs searched against the NCBI virus RefSeq database showed that SMV and CPMMV were widely found in these samples. Most samples (8/11) were co-infected by SMV and CPMMV, and the remaining three samples (AH_FY, HN_XC and HB_JZ) were infected by CPMMV alone (Table 1). These results were confirmed by reverse transcription PCR (RT-PCR) using primers specific for SMV or CPMMV (Supplementary Figure 1). These results indicate that CPMMV is distributed in several soybean producing areas in China and is commonly present in co-infections with SMV.

**TABLE 1:** Summary of Illumina sequencing and assembling statistics.

Location	Sample ID	Total reads	SMV			CPMMV		
				
			Acc. no.	Length (nt)	Alignment rate*	Acc. No.	Length (nt)	Alignment rate*
Anhui-Suzhou	AH_SZ	29.96 M	MW354946	9,990 bp	0.03%	MN908944	8,202 bp	23.07%
Anhui-Fuyang	AH_FY	32.56 M	–	–	–	MW354945	8,212 bp	18.23%
Shandong-Jiaxiang	SD_JX	54.50 M	MW354949	7,326 bp	1.09%	MW354937	8,202 bp	0.19%
Shandong-Jining	SD_JN	30.85 M	MW354953	7,137 bp	0.08%	MW354941	8,200 bp	0.11%
Henan-Luohe	HN_LH	41.61 M	MW354950	7,882 bp	0.12%	MW354936	8,200 bp	3.02%
Henan-Xuchang	HN_XC	28.70 M	–	–	–	MW354938	8,175 bp	0.24%
Henan-Shangqiu	HN_SQ	25.74 M	MW354952	7,167 bp	0.03%	MW354940	8,204 bp	4.22%
Jingsu-Nanjing	JS_NJ	30.23 M	MW354948	10,008 bp	43.11%	MW354944	8,338 bp	12.31%
Jilin-Changchun	JL_CC	45.80 M	MW354951	9,947 bp	18.61%	MW354943	8,212 bp	0.03%
Jilin-Gongzhuling	JL_GZL	26.38 M	MW354947	9,604 bp	21.09%	MW354942	8,172 bp	0.01%
Hubei-Jingzhou	HB_JZ	25.38 M	–	–	–	MW354939	8,178 bp	0.12%

*Virus-specific reads/reads.

### Phylogenetic Analysis of SMV and CPMMV Isolates

**FIGURE 2:**
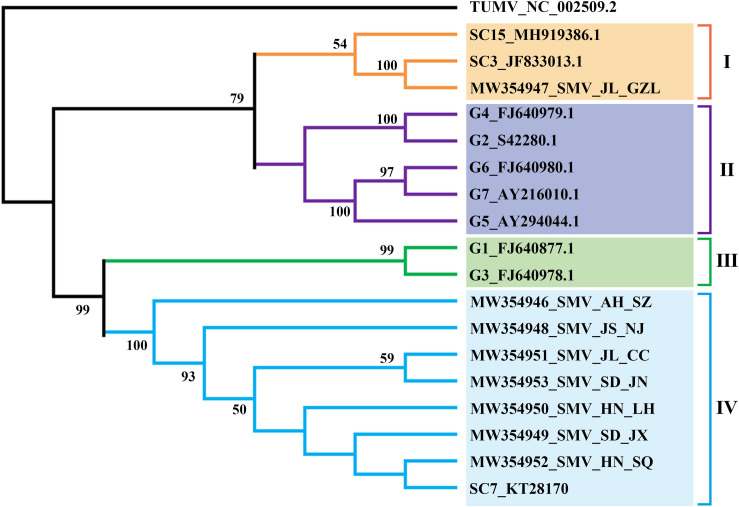
ML-phylogenetic relationships among soybean mosaic virus (SMV) isolates from different countries. The phylogenic tree was constructed using MEGAX. Near-complete genome sequences of SMV isolates were obtained from NCBI (https://www.ncbi.nlm.nih.gov/). The colored bars (blue, orange, green, purple) indicate the different clades observed in the phylogenies.

From the transcriptome sequencing data, the near-complete genome sequences of four SMV isolates and >7kb of four others (Supplementary Figure 2) were assembled and deposited in GenBank (Table 1). To determine the phylogenetic relationships between these eight SMV sequences and other known SMV genomes (G1-G7, United States; SC3, SC7 and SC15, the most important strains in China), we performed phylogenetic analysis based on the nucleotide sequence of the region from P3 to 6K2 using Maximum-Likelihood (ML) methods. The results showed four major clades and that the SMV isolates from China clustered separately from those of USA (Figure 2). Clade IV included all the new SMV isolates from this study (except SMV_JL_GZL from Jilin province) and the SC7 strain, while the isolate SMV_JL_GZL from northeast of China clustered with the SC3 strain. Clades II and III included the USA isolates G1–G7.

**FIGURE 3:**
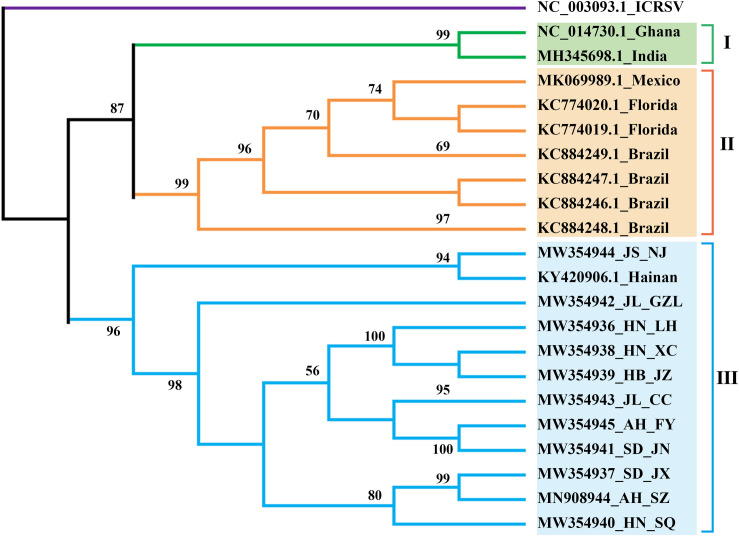
ML-phylogenetic analysis of the complete genomes of cowpea mild mottle virus (CPMMV) isolates from different countries. The phylogenic tree was constructed using MEGAX. Near-complete genome sequences of CPMMV isolates were obtained from NCBI (https://www.ncbi.nlm.nih.gov/). The colored bars (blue, orange, green) indicate the different clades observed in the phylogenies. Blue, China; Orange, Brazil and Florida; Green, India, and Ghana.

We assembled the complete CPMMV sequences from each of our 11 samples, deposited them in GenBank (Table 1), and examined the phylogenetic relationships between them and some representative complete CPMMV sequences from Brazil, United States, India, Mexico and Ghana. As shown in Figure 3, there were three lineages. The CPMMV isolates from our samples were closely related to each other within clade III and with a Chinese cowpea isolate (KY420906.1_Hainan). The CPMMV isolates from Brazil (soybean), Mexico (common bean) and Florida (whitefly) were placed in Clade II, while the isolates from Ghana and India were in clade I. Thus, the CPMMV isolates from China form a very distinct cluster. In phylogenetic analyses of each ORF separately, the same pattern of clustering was consistently obtained (Supplementary Table 1 and Supplementary Figure 3).

### Sequence Comparisons of CPMMV Isolates

Because the genetic diversity among CPMMV isolates has been little studied, sequence comparisons were made separately for each ORF of the CPMMV isolates. Among the isolates from China, the nucleotide identity of each ORF was in the range from 95 to 100% but the values were much lower when the Chinese isolates were compared with those from other countries (63.5–82.5%) (Supplementary Figure 4). ORF1 of the Chinese isolates all encoded 1,860 amino acids (Supplementary Table 1) and were very similar to one another (95.3–99.6% nt identity), but had 79.3–81.0% identity with Brazilian isolates and 75 and 64% identity with those from India and Ghana, respectively. In ORF2, the isolates collected in this study had 98.1–99.7% nucleotide identity to one another and the ORF was 11 codons shorter than that of the previously reported CPMMV isolate on cowpea from China. ORF3 and ORF4 were the same size among the isolates from China, Brazil, Florida and India, but not Ghana (Supplementary Table 1). ORF5 encodes the coat protein of 289 aa (Supplementary Table 1), and is the most conserved region among all isolates (Supplementary Figure 4). Among the isolates from China, there was 97.6–100% nt identity and 100% amino acid identity in this ORF, which had 78.3–78.9% nt identity with that of the isolate from Ghana. The ORF6 of Chinese isolates were all the same size and shared 94.9–100% nt identity, but only 59.4–88.5% identity isolates from India and Ghana (Supplementary Figure 4). Overall, our results confirm that the isolates from China are different from those reported in other countries, and represent a unique CPMMV strain.

### Recombination Analyses of CPMMV and SMV Isolates

**FIGURE 4:**
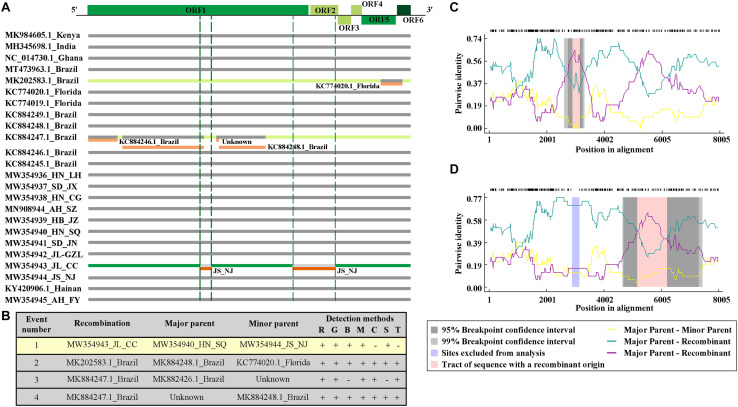
Recombination events among the CPMMV isolates. **(A)** Schematic representation of the recombination events identified among CPMMV isolates by the RDP 5 program. Each box represents a viral isolate with the recombination events identified by different color (green and orange). The CPMMV genome is shown at the top of the figure. **(B)** Details of recombination events in the complete coding sequence of CPMMV isolates. Recombination detection methods are represented by letters: R, RDP; G, Genecov; B, Bootscan; M, Maximum v2; C, Chimera; S, Sister scan; 3, 3 Seq. **(C,D)** Evidence of recombination event in the isolate JL_SMV1.

To detect possible recombination events amongst the CPMMV isolates, the complete coding sequences of all known CPMMV isolates in NCBI were analyzed using RDP 5. Four putative recombination events were detected among the isolates but only one among the Chinese isolates (Figure 4A). The isolates CPMMV_HN-SQ and CPMMV_JS-NJ were identified as the respective major and minor parents of CPMMV_JL_CC in a single recombination event identified in the region from nt 2,164 to 3,308 in ORF1 (Figures 4A–C). The beginning and ending breakpoints were identified at nt 2,891 and 3,146. This result was supported by five methods (RDP, *p* = 2.022 × 10^–05^; Geneconv, *p* = 5.858 × 10^–04^; Bootscan, *p* = 1.931 × 10^–05^; MAXchi, *p* = 2.586 × 10^–02^; SiScan, *p* = 2.781 × 10^–04^) with high confidence and phylogenetic analysis of the putative recombinant and non-recombinant portions also support the conclusion (Supplementary Figure 5).

We also detected six recombination events in the SMV population using the nucleotide sequence covering the region of the genome from P3 to 6K2. Two recombination events occurred in the SMV isolates we identified in this study. Recombination event 1 showed SC7 as the putative major parent and SMV_JL_GZL as the minor parent, which led to recombinant isolate SMV_AH_SZ. SMV_JL_GZL was identified as anther recombinant isolate, with G4 and isolate SMV_AH_SZ as possible parents (Supplementary Figure 6). This result was supported by four to six methods with high confidence.

### Symptoms on Mechanically Inoculated Plants

**FIGURE 5:**
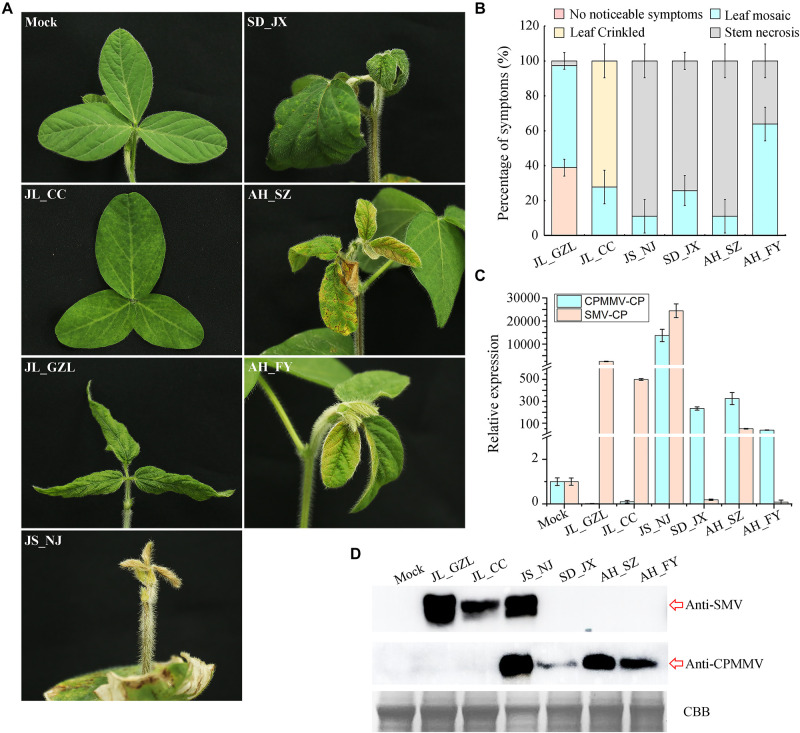
Symptoms of SMV/CPMMV infection on soybean. **(A)** Photographs of soybean (Jiunong 9) pants 10 days after inoculation with sap from different field locations. **(B)** The percentages of SMV/CPMMV-infected plants with different grades of disease symptoms, *n* = 12. **(C)** SMV and CPMMV RNAs in infected plants as determined by RT-qPCR. Data are shown as means ± *SD* of three biological replicates. **(D)** SMV and CPMMV coat protein (CP) protein levels in mock-inoculated and SMV/CPMMV-infected plants as determined by immunoblot analysis. Coomassie brilliant blue (CBB) staining of the same extracts is shown to demonstrate equal loading.

To further characterize the symptoms of CPMMV/SMV caused by the different isolates studied, the samples were used for mechanical transmission assays on susceptible soybean variety “Jiunong 9.” Successful infection was established following inoculation by seven of the mixed sap samples (three to five symptomatic plants) from each field, with systemic symptoms including stem necrosis, mosaic, and crinkled leaves appearing from 7 to 14 days post-inoculation (dpi) (Figure 5A). Most of the plants inoculated with JL_GZL (8.67 ± 1.15/12) developed leaf crinkling while the other plants had leaf mosaic (3.33 ± 1.15/12). About 60% of plants inoculated with sample JL_CC became infected, mainly showing mosaic symptoms (7.00 ± 0.00/12) (Figures 5A,B). RT-qPCR and western-blotting detected RNA and coat protein (CP) of SMV but not CPMMV in these plants (Figures 5C,D) even though the field samples had been co-infected with both viruses. By contrast, plants inoculated with sample SD_JX, developed leaf mosaic (3.33 ± 0.58/12) or stem necrosis (8.67 ± 0.58/12) (Figures 5A,B) and the RT-qPCR and immunoblot analyses showed that CPMMV, but not SMV, was present in the inoculated plants. Inoculation with either JS_NJ or AH_SZ, resulted in co-infection by CPMMV and SMV (Figures 5C,D) with mosaic symptoms on the upper leaves at the early infection stages, followed by systemic necrosis throughout the plants (Figure 5B). HTS results had shown that field sample AH_FY contained only CPMMV (Table 1). Most of the plants mechanically inoculated with AH_FY developed mild stem necrosis symptoms (7.67 ± 1.15/12), while the remainder had leaf mosaic (4.33 ± 1.15/12).

### Co-infection of SMV and CPMMV Decreased the Accumulation of CPMMV

**FIGURE 6:**
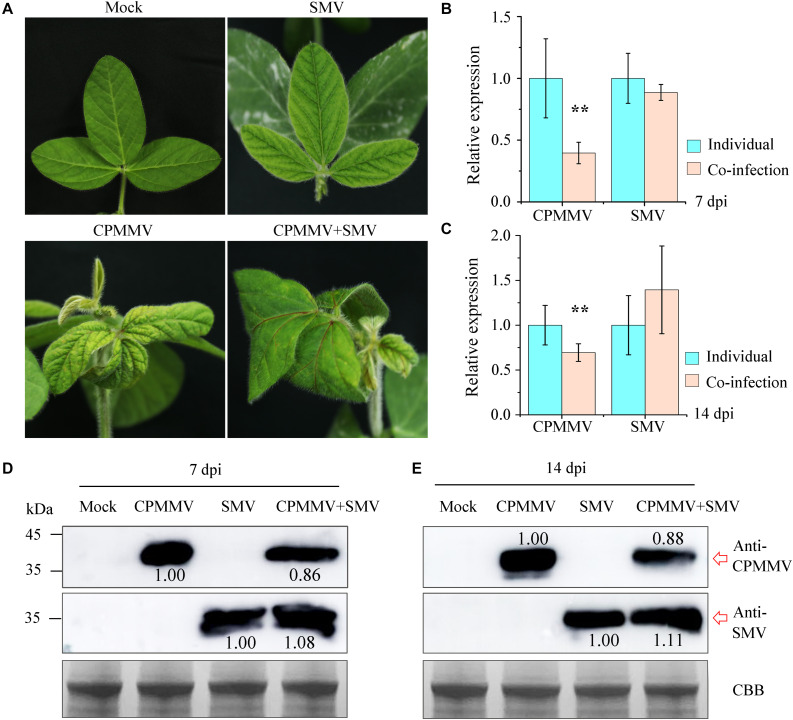
Co-infection of CPMMV and SMV increased the accumulation of SMV. **(A)** Symptoms on the first newly-emerged leaves 7 days after inoculation with SMV, CPMMV or SMV+CPMMV. **(B,C)** The relative expression levels of SMV and CPMMV RNAs as determined by RT-qPCR at 7 dpi **(B)** and 14 dpi **(C)**. Data are shown as means ± *SD* of three biological replicates, **P < 0.01, Student’s *t*-tests. **(D,E)** SMV and CPMMV coat protein (CP) protein levels in mock-inoculated and SMV/CPMMV-infected plants as determined by immunoblot analysis at 7 dpi **(D)** and 14 dpi **(E)**. Coomassie brilliant blue (CBB) staining of the same extracts is shown to demonstrate equal loading.

To understand the interaction between CPMMV and SMV in co-infection, the sap from mechanically inoculated leaves of isolates AH_FY (CPMMV) and JL_GZL (SMV) were inoculated onto soybean seedlings separately or together. Control (mock) plants were inoculated with phosphate buffer. The plants singly infected with CPMMV or SMV developed mild stem necrosis or mosaic, respectively, at 7 dpi (Figure 6A), while co-infection caused more severe symptoms: systemically infected leaves became crinkled at 5 dpi and developed systemic necrosis at 7 dpi (Figure 6A). RT-qPCR (Figures 6B,C) and western-blotting (Figures 6D,E) at 7 and 14 dpi confirmed the presence of both viruses. Compared to plants inoculated with a single virus, the accumulation of CPMMV genomic RNA and of CP were decreased in co-infected leaves but there were no significant effects on the levels of SMV RNA or CP.

## Discussion

CPMMV was first discovered on cowpea in Ghana in 1973 (Brunt and Kenten, 1973), and subsequently found infecting soybeans in Thailand and the Ivory Coast in the 1980s (Iwaki, 1982; Thouvenel et al., 1982). CPMMV was considered of minor importance until the occurrence of outbreaks in soybean across Brazil in the 2000s (Zanardo et al., 2014a). CPMMV is transmitted by the widespread whitefly *Bemisia tabaci*. It induces stem necrosis symptoms, and can cause economic losses in soybean production (Jossey, 2013; Zanardo and Carvalho, 2017). There are few studies on CPMMV: of the 16 complete genomic sequences of CPMMV available in GenBank, most (8/16) are from Brazil. The complete sequences of two CPMMV isolates from China have previously been reported, one from cowpea in Hainan (Yang et al., 2019), and our recent study providing the first report of CPMMV infecting soybean in China showing that it was the cause of leaf mosaic and crinkling symptoms (Wei et al., 2020). We have now demonstrated that CPMMV is widespread in the soybean producing areas in China and was present in all 11 samples collected. Their genomic sequences were obtained through HTS and phylogenetic analysis showed that all the Chinese isolates form a distinct cluster with 95–100% nucleotide identity to one another but rather distantly related to CPMMV isolates from other countries. In the family *Betaflexiviridae*, viruses are classified in the same species if their CP or replicase proteins have more than 72% nt or 80% aa identity (Adams et al., 2012). The ORF1 (RdRp) and CP of isolates from China have 79.3–81.0% and 83.0–90.0% nt identity to Brazilian isolates and clearly belong to the same species.

In Brazil, CPMMV causes a significant threat to soybean production and has received particular attention (Zanardo and Carvalho, 2017). The molecular variability of Brazilian CPMMV isolates has been investigated and it was concluded that the topology of the phylogenetic tree was not related to the geographical origin of isolates within Brazil (Zanardo et al., 2014a,b). Although the Chinese isolates form a separate branch of the CPMMV tree, there was similarly no evidence of further sub-clades based on their origin within China. This may suggest that CPMMV is a relatively recent introduction in both Brazil and China, or perhaps that the vector is able to rapidly distribute virus variants within the country. In addition, our phylogenetic analysis of SMV isolates also did not show clear relationships with their geographical origin. All SMV isolates (except SMV_JL_GZL from Jilin province) of this study were clustered in one clade with SC7 strain, and distinct from the US strains (G1–G7) (Figure 2). Recombination may explain the position of the SMV_JL_GZL isolate (Supplementary Figure 6). Recombination events have been identified in several viruses (Gagarinova et al., 2008; Zanardo et al., 2014b; Kalyandurg et al., 2017; Chikh-Ali et al., 2019). Our analysis detected one recombination event within the CPMMV isolates and two recombination events within the SMV isolates from China (Figure 4 and Supplementary Figure 6), indicating that recombination is one of the most important factors that contribute to the variability and evolution of SMV and CPMMV.

Previous studies have suggested that virus epidemics in soybean production in China are mainly caused by SMV and co-infection of soybean by SMV and CPMMV has not previously been reported. Our attempts to transmit virus from the co-infected field samples by mechanical inoculation sometimes led to the loss of one of the viruses. The mechanisms of co-infection are diverse in different host-pathogen systems (Bance, 1991; Gonzalez-Jara et al., 2004). Several lines of biochemical and genetic evidence have shown that viral interaction patterns in co-infection may depend on the host cultivar. For example, Wheat streak mosaic virus and Triticum mosaic virus induced cultivar-specific disease synergism in three wheat cultivars (Tatineni et al., 2010). We suppose that the different outcomes of mechanical inoculation in our study may be a consequence of differences in the virus content of field samples and host cultivar-pathogen specificity.

Co-infections by two or more viruses are common in the field. Although co-infections can be either synergistic or antagonistic (Syller, 2012; Bian et al., 2020), they usually cause more severe symptoms and significant damage to crops (Malapi-Nelson et al., 2009; Mahuku et al., 2015; Redinbaugh and Stewart, 2018). In our case, field observation and seedling inoculations suggest that co-infection causes more severe symptoms. However, at the molecular level, there would appear to be some antagonism between the viruses since RT-qPCR and western-blotting results showed that the accumulation level of CPMMV genomic RNAs and CP were decreased in co-infected leaves. This suggests that in co-infection with SMV, CPMMV interacts antagonistically. Similar observations have been reported with SMV and AMV infection in soybean, which showed severe symptoms in doubly infected plants, but the level of SMV accumulation was reduced. Conversely, the level of AMV accumulation was increased indicating that the interaction of AMV with SMV is synergistic (Malapi-Nelson et al., 2009). In the current study, there was no significant effect on the accumulation level of SMV in co-infected leaves. There appears to be a complex mechanism of interaction between SMV and CPMMV that requires further investigation.

## Data Availability Statement

The datasets presented in this study can be found in online repositories. The sequences of CPMMV and SMV isolates were deposited in The National Center for Biotechnology Information (NCBI) GenBank, https://www.ncbi.nlm.nih.gov/genbank/ and the accession numbers can be found in the article/Supplementary Material. The other raw data supporting the conclusions of this article are available upon request from the corresponding author, without undue reservation.

## Author Contributions

ZW and ZS designed the research and performed the experiments. ZW drafted the manuscript. ZS revised the manuscript. CM and CJ supervised the project. All authors contributed to the article and approved the submitted version.

## Conflict of Interest

The authors declare that the research was conducted in the absence of any commercial or financial relationships that could be construed as a potential conflict of interest.

## Footnotes

1 https://www.boldsystems.org/

2 ftp://ftp.ncbi.nlm.nih.gov/refseq/release/viral

3 https://www.ncbi.nlm.nih.gov/

## Supplementary Material

Supplementary Figure 1 Detection of CPMMV and SMV in the total RNA of symptomatic field soybean samples as shown by RT-PCR.

Supplementary Figure 2 The genome organization of SMV isolates.

Supplementary Figure 3 NJ-phylogenetic relationships among the ORFs of CPMMV isolates from different countries.

Supplementary Figure 4 Pairwise identity matrix of CPMMV isolates for each open reading frame (ORF).

Supplementary Figure 5 Phylogenetic trees analysis based on different nucleotide fragments of CPMMV isolates from China.

Supplementary Figure 6 Recombination events among the SMV isolates.

Supplementary Table 1 Genomic organization of the 11 CPMMV isolates described in this study.

Supplementary Table 2 List of primer sequences used in this study.
