# Supplementary material for: Identification of a New Genetic Clade of Cowpea Mild Mottle Virus and Characterization of Its Interaction With Soybean Mosaic Virus in Co-infected Soybean
Source: Front Microbiol. 2021 Apr 8;12:650773. doi: 10.3389/fmicb.2021.650773 (PMC8060446; doi:10.3389/fmicb.2021.650773)
Supplement: Supplementary Figure 1 — Detection of CPMMV and SMV in the total RNA of symptomatic field soybean samples as shown by RT-PCR. [file Data_Sheet_1.docx]

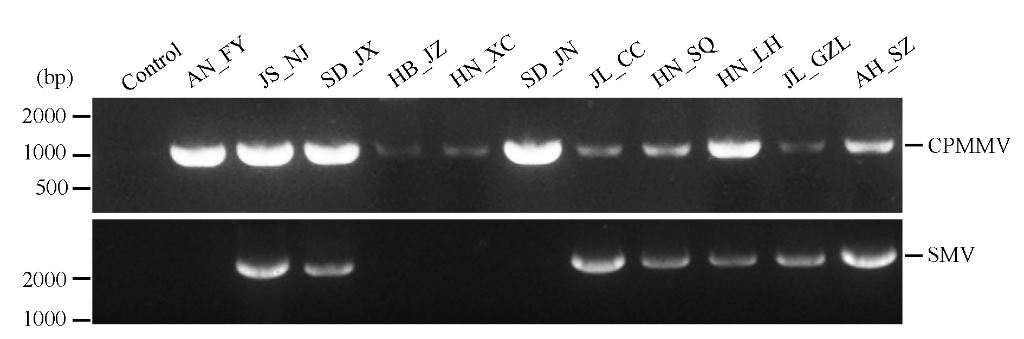


Supplementary Figure 1 Detection of CPMMV and SMV in the total RNA of symptomatic field soybean samples as shown by RT-PCR.


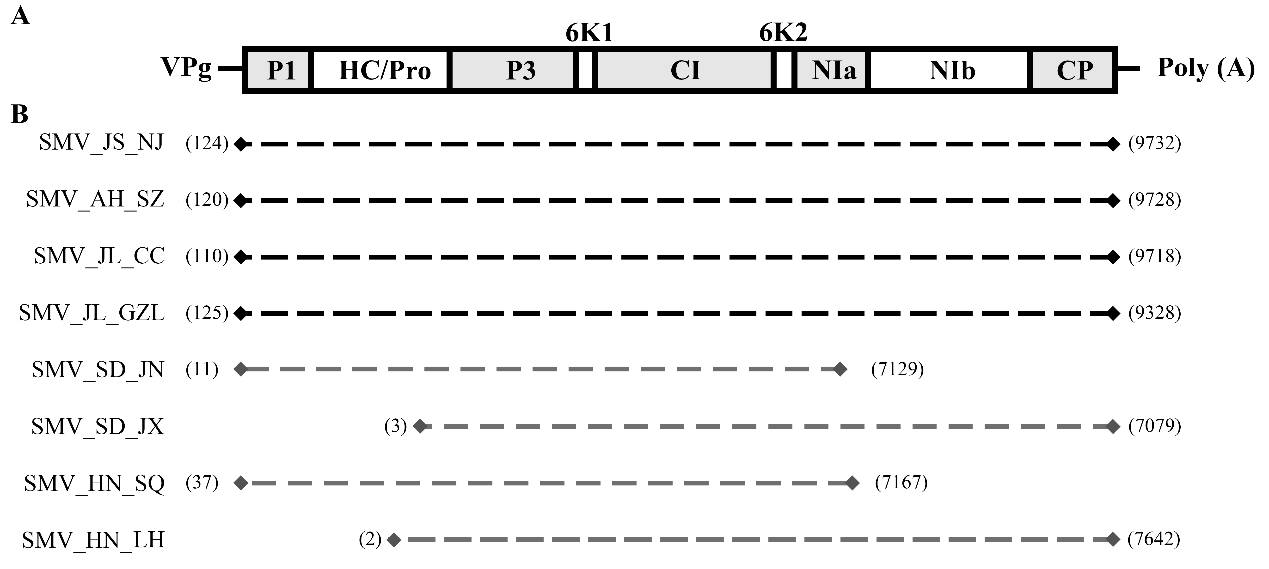


Supplementary Figure 2 The genome organization of SMV isolates

(A) Genome graph depicting the SMV genome organization. (B) Diagram of the assembled SMV sequences in this study


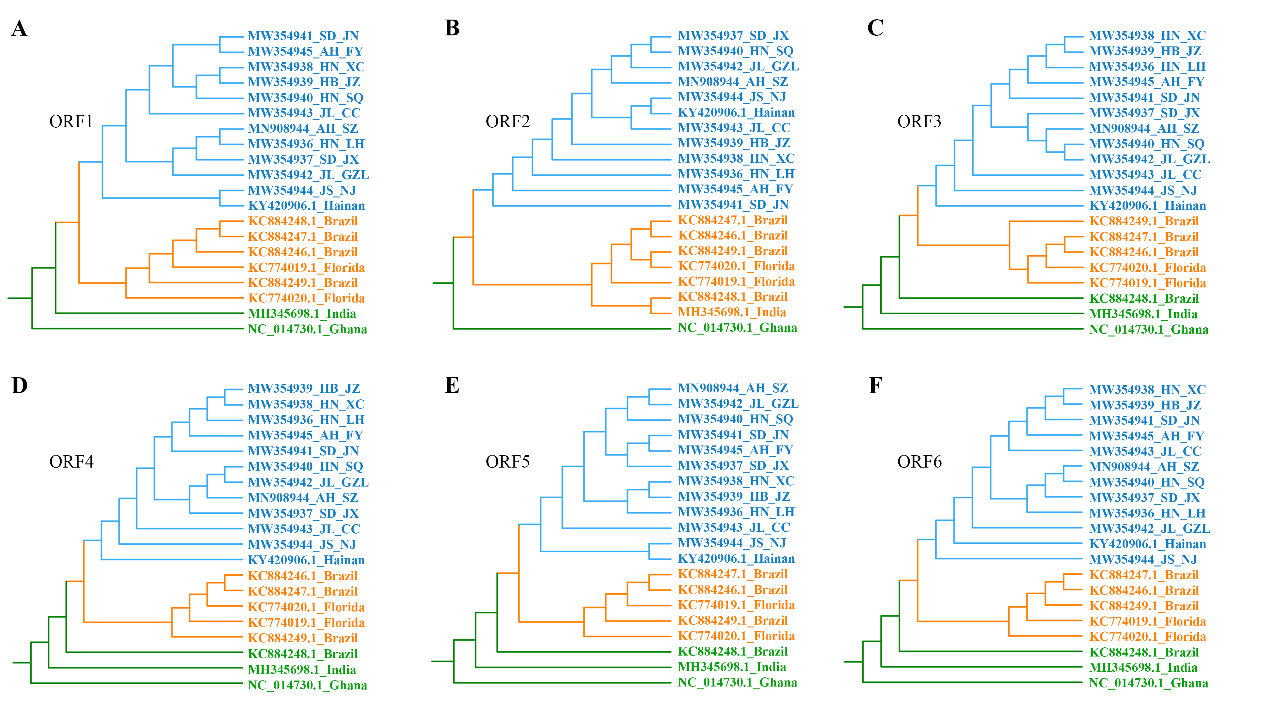


Supplementary Figure 3 NJ-phylogenetic relationships among the ORFs of CPMMV isolates from different countries.

The phylogenic tree was constructed using MEGA version 5.1. (A) ORF1, RNA-dependent RNA polymerase (RdRp); (B-D) ORFs 2–4, triple gene block (TGB1, TGB2 and TGB3); (E) ORF 5, coat protein (CP); (F) ORF6, nucleic acid-binding protein (NABP).


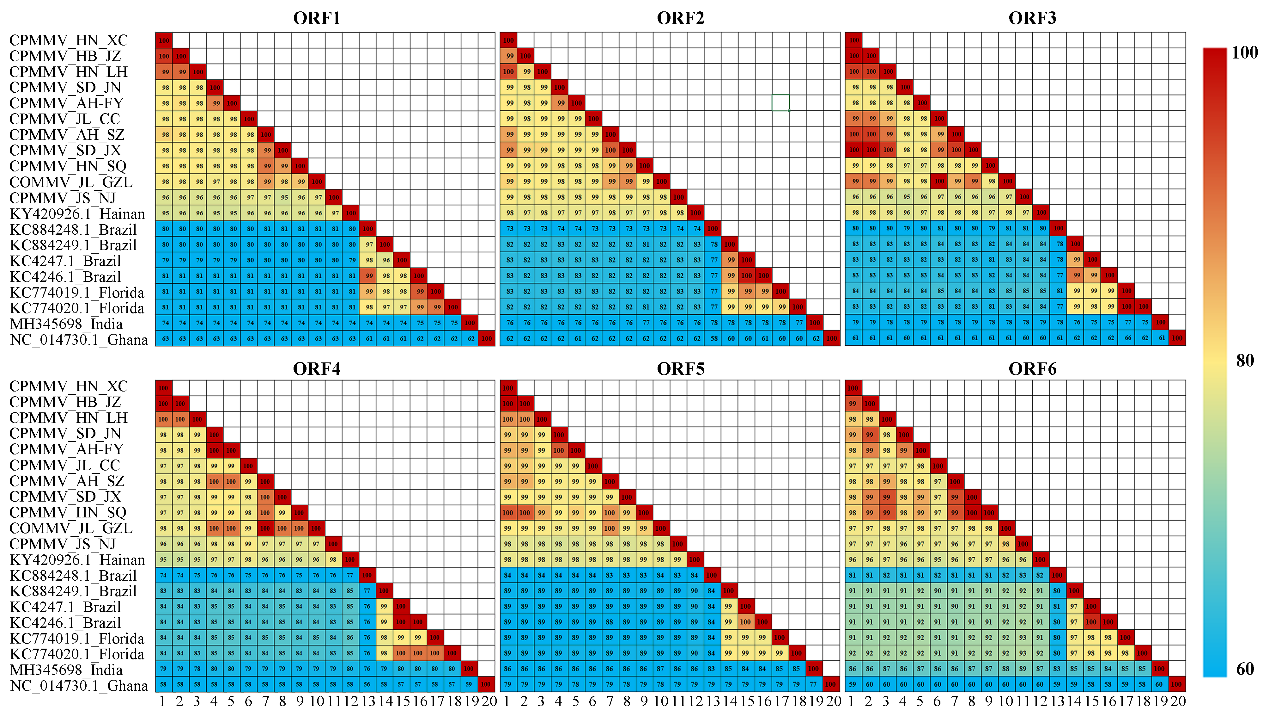


Supplementary Figure 4 Pairwise identity matrix of CPMMV isolates for each open reading frame (ORF)

Percentage nucleotide sequence identities are shown below the diagonal. ORF1, RNA-dependent RNA polymerase (RdRp); ORFs 2–4, triple gene block (TGB1, TGB2 and TGB3); ORF 5, coat protein (CP); ORF6, nucleic acid-binding protein (NABP).


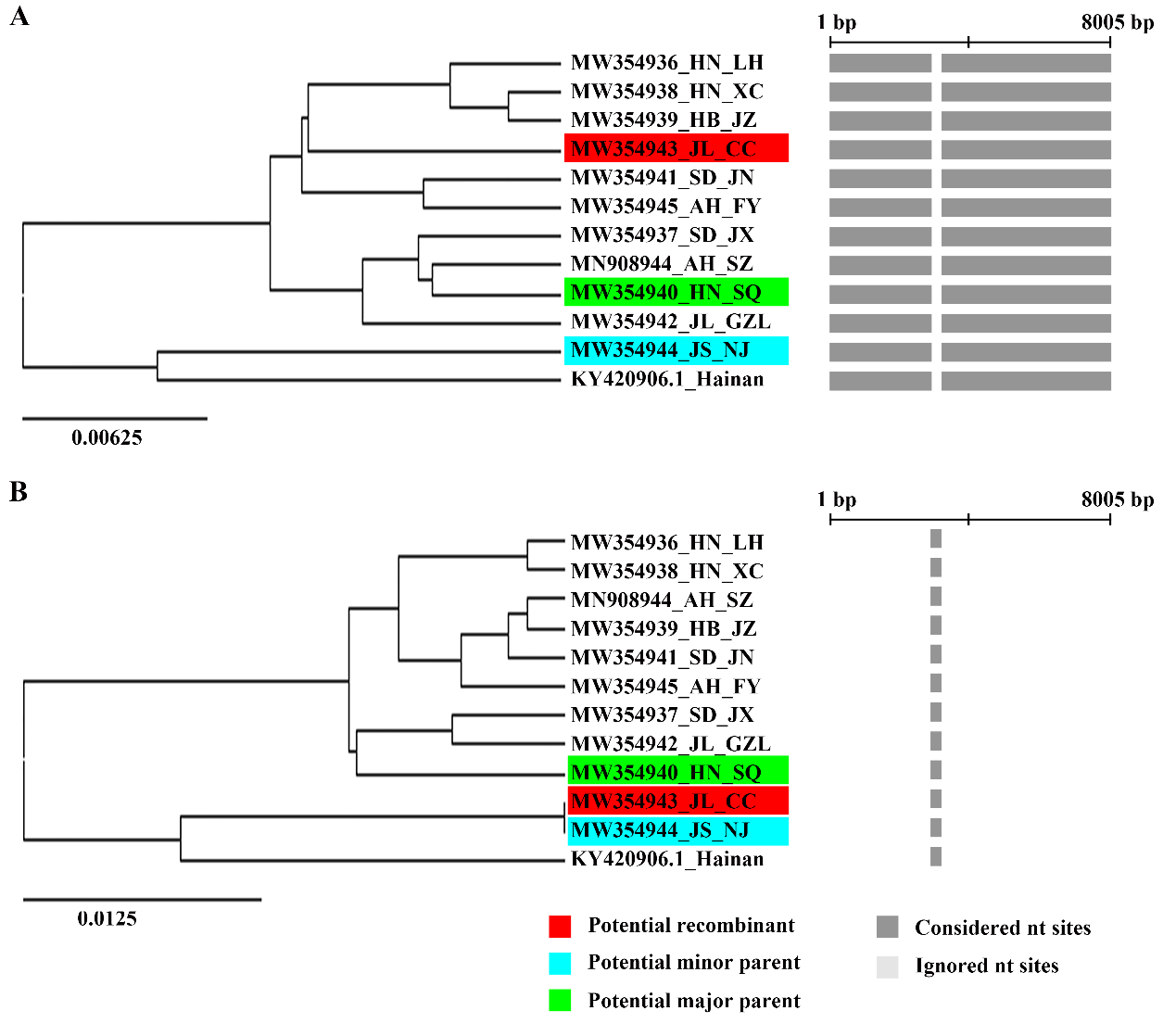


Supplementary Figure 5 Phylogenetic trees analysis based on different nucleotide fragments of CPMMV isolates from China.

Phylogenetic trees were constructed using different nucleotide fragments based on the results of the recombination analysis. (A) UPGMA of regions derived from major parent (1-2890 and 3147-8005 nt). (B) UPGMA of regions derived from minor parent (2891-3146 nt).

**
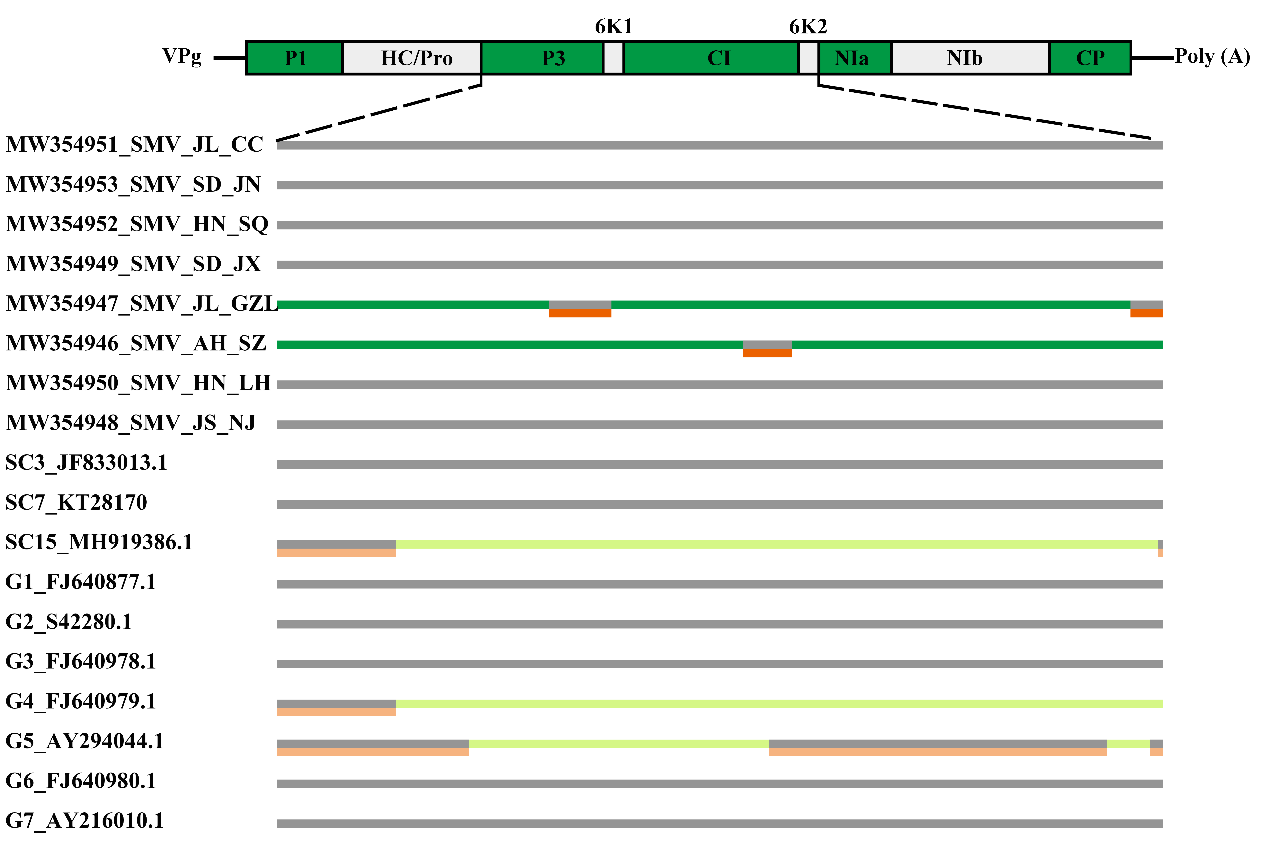
**

Supplementary Figure 6 Recombination events among the SMV isolates

Schematic representation of the recombination events identified among SMV isolates by the RDP 5 program. Each box represents a viral isolate with the recombination events identified by different color (green and orange). The SMV genome is shown at the top of the figure.

**Supplementary Table 1** Genomic organization of the 11 CPMMV isolates described in this study

| Isolates | 5’-UTR | RP | | TGB1 | | TGB2 | | TGB3 | | NABP | | CP | | 3’-UTR |
| --- | --- | --- | --- | --- | --- | --- | --- | --- | --- | --- | --- | --- | --- | --- |
|  | #nt | nt position | #aa | nt position | #aa | nt position | #aa | nt position | #aa | nt position | #aa | nt position | #aa | #nt |
| HN-LH | 71 | 72-5651 | 1860 | 5680-6375 | 232 | 6375-6695 | 107 | 6673-6879 | 69 | 6895-7761 | 289 | 7764-8075 | 104 | 134 |
| SD-JX | 71 | 72-5651 | 1860 | 5680-6375 | 232 | 6375-6695 | 107 | 6673-6879 | 69 | 6895-7761 | 289 | 7764-8075 | 104 | 138 |
| AH-TH | 73 | 74-5653 | 1860 | 5682-6377 | 232 | 6377-6697 | 107 | 6675-6881 | 69 | 6897-7763 | 289 | 7766-8077 | 104 | 135 |
| HN-CG | 61 | 62-5641 | 1860 | 5670-6365 | 232 | 6365-6685 | 107 | 6663-6869 | 69 | 6885-7751 | 289 | 7754-8065 | 104 | 110 |
| AH-SZ | 62 | 63-5642 | 1860 | 5671-6366 | 232 | 6366-6686 | 107 | 6664-6870 | 69 | 6886-7752 | 289 | 7755-8066 | 104 | 126 |
| HB-JZ | 60 | 61-5640 | 1860 | 5669-6364 | 232 | 6364-6684 | 107 | 6662-6868 | 69 | 6884-7750 | 289 | 7753-8064 | 104 | 100 |
| HN-SQ | 74 | 75-5654 | 1860 | 5683-6378 | 232 | 6378-6698 | 107 | 6676-6882 | 69 | 6898-7764 | 289 | 7767-8078 | 104 | 126 |
| SD-JN | 71 | 72-5651 | 1860 | 5680-6375 | 232 | 6375-6695 | 107 | 6673-6879 | 69 | 6895-7761 | 289 | 7764-8075 | 104 | 125 |
| JL-SMV3 | 68 | 69-5648 | 1860 | 5677-6372 | 232 | 6372-6692 | 107 | 6670-6876 | 69 | 6892-7758 | 289 | 7761-8072 | 104 | 100 |
| JL-SMV1 | 64 | 65-5644 | 1860 | 5673-6368 | 232 | 6368-6688 | 107 | 6666-6872 | 69 | 6888-7754 | 289 | 7757-8068 | 104 | 144 |
| NJ-JS | 75 | 76-5655 | 1860 | 5684-6379 | 232 | 6379-6699 | 107 | 6677-6883 | 69 | 6899-7765 | 289 | 7768-8079 | 104 | 162 |
| Hainan | 72 | 73-5652 | 1860 | 5681-6409 | 243 | 6375-6695 | 107 | 6673-6879 | 69 | 6895-7761 | 289 | 7764-8075 | 104 | 118 |
| KC884248.1_Brazil | 72 | 73-5652 | 1860 | 5681-6385 | 235 | 6376-6696 | 107 | 6674-6880 | 69 | 6895-7761 | 289 | 7764-8162 | 133 | 36 |
| KC774020.1_Florida | 71 | 72-5651 | 1860 | 5680-6375 | 232 | 6375-6695 | 107 | 6673-6879 | 69 | 6895-7761 | 289 | 7764-8105 | 114 | 104 |
| MH345698.1_India | 69 | 70-5652 | 1861 | 5681-6376 | 232 | 6376-6696 | 107 | 6674-6880 | 69 | 6895-7761 | 289 | 7764-8075 | 104 | 114 |
| Ghana | 73 | 74-5584 | 1837 | 5613-6317 | 235 | 6308-6685 | 126 | 6606-6815 | 70 | 6833-7699 | 289 | 7702-8010 | 103 | 117 |

**Supplementary Table 2** List of primer sequences used in this study

| **Usage** | **Primer Name** | **Sequence** |
| --- | --- | --- |
| Quantitative RT-PCR | CPMMV-CP-F | 5′ TCAAATAACATGGCCACAGC 3′ |
|  | CPMMV-CP-R | 5′ GCAACCTGCAAAAGAACCTC 3′ |
|  | SMV-CP-F | 5′ GTAGCAAGGGAGCTGGAACA 3′ |
|  | SMV-CP -R | 5′ CTTCCCTTCAACCATTGGAA 3′ |
|  | Actin11-F | 5′ CGGTGGTTCTATCTTGGCATC 3′ |
|  | Actin11-R | 5′ GTCTTTCGCTTCAATAACCCTA 3′ |
| Detection  RT-PCR | CPMMV-F | 5′ CGATCCTGTCGAGATTGGTT 3′ |
|  | CPMMV-R | 5′ AGAAGGCCCTCAAATCATCC 3′ |
|  | SMV-F | 5′ CGGAACTGCTATGCAGACAA 3′ |
|  | SMV-R | 5′ TTAGAGGACTGTCCCCAAGT 3′ |
